# Root-Associated Microbiota Response to Ecological Factors: Role of Soil Acidity in Enhancing Citrus Tolerance to Huanglongbing

**DOI:** 10.3389/fpls.2022.937414

**Published:** 2022-07-15

**Authors:** Bo Li, Yanan Wang, Tongle Hu, Dewen Qiu, Frédéric Francis, Shuangchao Wang, Shutong Wang

**Affiliations:** ^1^State Key Laboratory of North China Crop Improvement and Regulation, College of Plant Protection, Hebei Agricultural University, Baoding, China; ^2^Department of Functional and Evolutionary Entomology, Gembloux Agro-Bio Tech, University of Liège, Gembloux, Belgium; ^3^The State Key Laboratory of Plant Diseases and Insect Pests, Institute of Plant Protection, Chinese Academy of Agricultural Sciences, Beijing, China

**Keywords:** *Candidatus* Liberibacter, soil acidification, metagenomics, endophyte, rhizoplane, defense response, plant-microbe interactions

## Abstract

The citrus orchards in southern China are widely threatened by low soil pH and Huanglongbing (HLB) prevalence. Notably, the lime application has been used to optimize soil pH, which is propitious to maintain root health and enhance HLB tolerance of citrus; however, little is known about the interactive effects of soil acidity on the soil properties and root-associated (rhizoplane and endosphere) microbial community of HLB-infected citrus orchard. In this study, the differences in microbial community structures and functions between the acidified and amended soils in the Gannan citrus orchard were investigated, which may represent the response of the host-associated microbiome in diseased roots and rhizoplane to dynamic soil acidity. Our findings demonstrated that the severity of soil acidification and aluminum toxicity was mitigated after soil improvement, accompanied by the increase in root activity and the decrease of HLB pathogen concentration in citrus roots. Additionally, the Illumina sequencing-based community analysis showed that the application of soil amendment enriched functional categories involved in host-microbe interactions and nitrogen and sulfur metabolisms in the HLB-infected citrus rhizoplane; and it also strongly altered root endophytic microbial community diversity and structure, which represented by the enrichment of beneficial microorganisms in diseased roots. These changes in rhizoplane-enriched functional properties and microbial composition may subsequently benefit the plant's health and tolerance to HLB disease. Overall, this study advances our understanding of the important role of root-associated microbiota changes and ecological factors, such as soil acidity, in delaying and alleviating HLB disease.

## Introduction

Soil acidification has attracted widespread attention due to its negative impacts, including fertility degradation and potential production, and declined root growth of sensitive species. The acidification caused by excessive addition of nitrogen accelerates the loss of base cations, such as calcium (Ca), magnesium (Mg), potassium (K), and sodium (Na), which leads to soil fertility degradation (Zhu et al., 2018). The availability of these base cations is significantly limited because they are replaced by protons and subsequently leached from the root zone accompanied by nitrate in acidic soils (Yang et al., 2013). Therefore, soil pH probably affects plant growth and vitality by limiting nutrient bioavailability. On the other hand, the solubility of toxic aluminum (Al) and manganese (Mn) in highly acidic soil is greatly elevated, which may weaken the productivity of plants by stunting and injuring plant roots (Arunakumara et al., 2012; Riaz et al., 2018). According to previous studies, more than 70% of acidic soils exhibit Al-toxicity and lack of Mg and Ca in tropical America (George et al., 2011). The above indications indicate that the acidified soils can act as a constraint on plant health and disease resistance/tolerance.

Soil is the largest known microbial diversity pool (Torsvik et al., 2002), and rhizoplane are the primary site for plant-microbe interactions. Normally, soil acidity is considered to exert a powerful environmental filter on the soil microbial community assembly and control the activities of soil microbes in surface soils (Schlatter et al., 2020). Meanwhile, the soil microbes, especially bacteria, are key components of the underground ecosystem, which contribute to plant health and defense by affecting carbon and nutrient cycling, plant growth promotion, pesticide degradation, and protecting plants from pathogen infection (Zhu et al., 2014; Trivedi et al., 2020). However, soil acidification weakens and even ceases the activity and survival of beneficial soil organisms, such as nitrogen fixers, decomposers, and nutrient recyclers (Jacoby et al., 2017). On the other hand, the successful colonization and invasion of some soil-borne pathogens are closely related to the ambient pH levels, which regulate the synthesis of pathogenesis factors and the expression of virulence and survival-related genes (Manteau et al., 2003). In South China, soil acidification aggravates the occurrence of bacterial wilt in tobacco plants, because acidic conditions (pH 4.5–5.5) favored the growth of the pathogen *Ralstonia solanacearum*, but inhibited the growth of antagonistic bacteria of *Pseudomonas fluorescens* and *Bacillus cereus* (Li et al., 2017). Altogether, many studies have reported that decreased pH has a direct influence on microbial structure and leads to the imbalance in the topsoil micro-ecosystem and even the accumulation of soil-borne pathogens in acidified soil. However, up to now, researchers have paid limited attention to the correlation between soil acidification and systemic diseases with root symptoms caused by non-soil-borne pathogens, and the impact of soil acidification on the structure and function of the soil microbial community of plants infected with systemic diseases.

Huanglongbing (HLB) is a systemic disease of citrus caused by phloem-limited and nonculturable bacteria “*Candidatus* Liberibacter” spp., which is the greatest threat to citrus production worldwide. In south China, HLB is associated with Asian strain “*Ca*. L. asiaticus” (CLas) is transmitted by citrus psyllids to all commercial cultivars of citrus., the above-ground symptoms of HLB in orchards include blotchy mottle, and yellow shoots, and asymmetric fruits (Li et al., 2020). Inexplicably, the discrepancy between the slow development of foliar symptoms and the rapid yield losses of HLB-infected citrus trees, indicates that unobserved underground damage, such as root decline, has occurred before the emergence of foliar symptoms (Johnson et al., 2014). Meanwhile, the root damage and lower fibrous root density of the infected trees could restrict the absorption of water and soil nutrients (Atta et al., 2020). Additionally, HLB invasion has a non-negligible effect on the remodeling of citrus root-associated microbes, which causes enrichment and reduction of specific disease-induced root-associated microbial taxa in the early and late phases of HLB disease (Trivedi et al., 2010, 2012; Zhang et al., 2017; Ginnan et al., 2020). As one of the top three fruit crops in the world, the citrus industry has developed rapidly in China in recent years. Unfortunately, HLB has emerged in 10 of China's 11 major citrus-growing provinces, but there was no definite and sustainable cure insight (Zhou, 2020). The global citrus industry is facing the huge challenge of HLB invasion, and the prevention and control situation is still grim.

Another challenge of citrus planting in China is soil acidification and nutrient deficiencies. It is well known that citrus cannot thrive in acidic soils, especially when the soil pH is 5.0 or lower, serious problems may arise, which make citrus more susceptible to abiotic and biotic stresses (Long et al., 2017). Regrettably, most of the citrus orchards in China have moderately or strongly acidic soils. Fan et al. (2015) reported that the pH values of over 1,400 soil samples from 477 orchards in 18 counties of Ganzhou, Jiangxi Province was 4.6, of which 80.0% of orchard soils with a pH value lower than 5.0. Citrus plants are perennials, and their roots are exposed to the same soil during their life cycle. Therefore, it is necessary to maintain proper soil acidity in the root zone to avoid Al-toxicity and provide sufficient calcium and magnesium for citrus growth (Natale et al., 2012). Moreover, the causal agents cannot be cultured, which is a great obstacle to revealing the direct interaction between HLB pathogen and ecological factors. Our previous study showed that the application of lime in the HLB-endemic and soil acidified citrus orchards could efficiently correct soil acidity and delay the progress of HLB, thereby improving the fruit yield and quality of citrus groves (Li et al., 2020). The correction of soil acidity enhanced the tolerance of citrus to HLB disease, which is due to the direct promotion of citrus root metabolic activity and the direct activation of citrus immune defense response. On this basis, we also speculate that this transformation in HLB tolerance may be related to the reshaping of citrus root and rhizoplane microbiome. Therefore, more studies are required to explore how soil acidification affects the microbial community assembly process of the HLB-infected citrus root-associated microbiome.

In this study, we evaluated the effects of lime treatment on the soil properties and the resident microbial community of HLB-infected citrus roots in acidic soil. We combined lime application and microbiological sequencing analysis to investigate the distinct and enriched taxa and functions of citrus root and rhizoplane, and how soil acidity affects those enriched taxonomic and functional attributes in HLB root-related microbiomes. Our results emphasize the associations, not causation, between HLB pathogen, microbiota, soil acidity, and citrus tolerance.

## Materials and Methods

### Site Description and Experiment Set Up

This study was performed in a citrus orchard in Xunwu Country, Ganzhou City, Jiangxi Province, China (lat 24°94′ N, long 115°44′ E) in 2019. The region is characterized by a subtropical, humid monsoon climate with a mean annual rainfall of 1,600 mm and an average annual temperature of 18.9°C. The soil at this site belongs to clay red soil, which is derived from the argillaceous rock with high soil acidity, and the average pH value is 4.3. The experimental plants were 8-year-old orchard trees of “Newhall” (*Citrus sinensis* Osbeck cv. Newhall) navel orange grafted on Carrizo citrange rootstock [*Citrus sinensis* (L.) Osb × *Poncirus trifoliate* (L.) Raf.], 2–3 m tall, and 1.5–2 m wide, spaced 3 × 4 m apart. In the experiment of lime application in citrus orchards, nine CLas-infected trees with equal disease severity were selected based on quantitative PCR results of root samples and visual foliar symptoms of HLB in <30% of the canopy. Three treatments were included in this study: SCK (soil without lime treatment), SLL (soil treated with low-level lime, 1.25 tons/ha), and SHL (soil treated with high-level lime, 2.5 tons/ha), and each treatment with three replications. The slaked lime used in the study was locally purchased, the calcium hydroxide content was higher than 60%, and more than 90% of the material could pass through an 80-mesh sieve. The slaked lime was sprinkled onto the soil surface in the 4 × 4 m area based on the center of the citrus tree trunk to ensure that the corrective had adequate coverage and then was mixed thoroughly into the soil layer by plowing in January 2019. In addition, a total of 450 g of N, 150 g P_2_O_5_, and 450 g of K_2_O were added per plant per year, which was applied in four portions by digging a shallow furrow under the canopy of each tree. A single application of 5 kg peanut cake fertilizer per plant per year was also made. Meanwhile, irrigation, weed, and pest management in orchards were timely implemented according to the unified standard. Fertilizer inputs (N-P_2_O_5_-K_2_O) occurred in all plots, including the control treatment.

### Soil and Plant Sample Collection

Fibrous roots (~1.5-mm diameter) and corresponding soil samples were collected at a depth of 5–15 cm soil layers 1 week before the harvest of citrus fruits in November 2019. For each tree, the samples were collected from the four locations ~0.8-m away from the trunk and were, respectively, pooled together as one composite sample. Firstly, the loosely attached soil on the roots was acquired with gentle shaking for soil property analysis and was termed root-zone soil. The roots were subsequently placed in sterilized and pre-cooled PBS buffer, and the rhizosphere soil adhering to the roots was removed by hand-shaking for 2 min, and the rhizoplane soil was extracted by ultra-sonication as described by Zhang et al. (2017). Briefly, the roots from the previous step were sonicated twice with a 60 Hz cold Ultrasonic water bath, each time for 30 s, with an interval of 5 s. Finally, the rhizoplane soil was collected by centrifugation at 12,000 × g for 1 min at 4°C and stored at −80°C until DNA extraction. On the other hand, about 5 g of ultrasonically treated root of each sample was preserved at −80°C after being drained with sterile filter paper for nucleic acid extraction, and another part of 10 g was stored at 4°C to test root activity immediately, and the remaining was air-dried to test the physicochemical properties.

### Nucleic Acid Extraction

Total DNA from rhizoplane soils and fibrous root segments was, respectively, extracted using the DNeasy Plant Mini Kit and the DNeasy PowerSoil Pro Kit (Qiagen, Valencia, CA, USA) according to the manufacturer's protocol. DNA quality and quantity were assessed using a TBS-380 Mini-Fluorometer (Turner Biosystems, CA, USA) and a NanoDrop 1000 spectrophotometer (Thermo Scientific, Wilmington, DE, USA), respectively.

### Detection of HLB Pathogen

The q-PCR analysis for CLas titers of root samples was performed with primers and probe as previously described (Li et al., 2006). Additionally, 1 μl (40 ng) of fine root template DNA was used in a 20 μl qPCR reaction volume on an ABI7500 Real-Time PCR system using TaKaRa Ex Taq HS DNA Polymerase according to the manufacturer's instructions. The standard amplification protocol was 95°C for 30 s, followed by 40 cycles at 95°C for 5 s and 60°C for 34 s. All reactions were performed in triplicate. The number of CLas genomes in a reaction was calculated using a recombinant plasmid standard curve containing the target sequence.

### Root Activity Measurement

Determination of root activity was done according to the triphenyl tetrazolium chloride (TTC) reduction test. First, 0.5 g fresh root tip samples were immersed in 10 ml of TTC solution (0.04% in phosphate buffer at pH 6.8) for 1 h at 37°C. Then, the reaction was terminated by adding 2 ml 1 M H_2_SO_4_. The roots were drained and then homogenized in ethyl acetate and centrifuged at 12,000 × g for 5 min. Finally, the liquid phase was adjusted to a volume of 10 ml with ethyl acetate and the absorbance of the extract was recorded at 485 nm.

### Soil Property Analysis

All soil samples were ground and sieved before soil analysis. The detailed analytical methods for measuring edaphic properties, including soil pH and the contents of soil organic carbon (SOC), available nitrogen (AN), available phosphorus (AP), available potassium (AK), exchangeable magnesium (Mg^2+^), calcium (Ca^2+^), and aluminum (Al^3+^) are described in previous studies (Kachurina et al., 2000; Nelson and Sommers, 2018; Wan et al., 2020). Briefly, Soil pH was measured at a ratio of soil/water of 1:2.5 (m/v) using a digital pH meter; SOC was measured by loss-on-ignition method, and AN was determined using the alkali hydrolyzation procedure. AP and AK were extracted with 0.5 M sodium bicarbonate and 1 M ammonium acetate, respectively, and then measured with inductively coupled plasma optical emission spectrometry. The exchangeable cations (Ca^2+^ and Mg^2+^) were extracted using ion exchange resins and determined by atomic absorption spectrometry. The exchangeable Al^3+^ was extracted with neutral 1 mol L^−1^ KCl at a 1:10 soil/solution ratio and determined by titration with a 0.025 mol L^−1^ NaOH solution.

### Next-Generation Sequencing

DNA extracted from citrus roots was selected for 16S amplicon sequencing and DNA extracted from rhizoplane soils was used for shotgun metagenomic sequencing, respectively. The samples were sequenced on the Illumina System by Majorbio Bio-Pharm Technology Co., Ltd. (Shanghai, China). For the amplicon sequencing, the amplification of 16S DNA fragments was performed using primer pairs 799F-1391R and 799F-1193R covering the prokaryotic 16S rDNA V5-7 regions. After quality control, quantification, and normalization of the DNA libraries, 300-bp paired-end reads were generated from the Illumina MiSeq PE300 platform (Illumina, San Diego, CA, USA) according to the manufacturer's instructions with modifications for 16S amplicon analyses. Large-scale shotgun metagenome sequencing was performed on the Illumina NovaSeq platform (Illumina Inc., San Diego, CA, USA) using NovaSeq Reagent Kits, and each DNA shotgun sequencing sample contained 6 Gb of sequencing data. All sequencing data associated with this project have been deposited in the NCBI Short Read Archive database (Bioproject accessions: PRJNA835291 and PRJNA827960).

The data were analyzed using the free online platform, the MajorBio i-Sanger cloud platform (www.i-sanger.com). For amplicon data analysis, the poor quality reads were subjected to filter and trim using Trimmomatic software and then merged paired-end reads using FLASH software with the default setting (Magoc and Salzberg, 2011). High-quality remaining sequences were clustered into operational taxonomic units (OTUs) at 97% similarity using UPARSE (Edgar, 2013). Representative sequences of each OTU were classified by the RDP classifier (Wang et al., 2007), and compared with the SILVA database (Quast et al., 2013). The final output of the taxonomic assignment results reached a confidence level of ~70% in different taxonomic levels. The resulting raw OTUs table was filtered to remove OTUs with less than two sequences in a single sample, and the filtered OTU table was used as input for further analysis. For metagenomic data analysis, the raw reads were pre-processed to remove poor-quality reads and adaptor sequences. The clean filtered reads from all soil samples were assembled using megahit v1.03 (Li et al., 2015). Then, all sequences with an identity cutoff of 95% were clustered and reduced redundancy to construct the non-redundant gene categories (unigenes) using CD-HIT-est (Fu et al., 2012). To access the taxonomic annotation of the unigenes, the amino acid sequences were aligned against the NCBI microbial non-redundant protein (Nr) database using the DIAMOND software (blastp, E-value cut-off of 1 × 10^−5^) (Buchfink et al., 2014). The microbial-metabolic functions of the unigenes were predicted by blasting against the Kyoto Encyclopedia of Genes and Genomes (KEGG) and Gene Ontology (GO) database. Moreover, the high-quality reads from rhizoplane samples were aligned against the unigenes to calculate the taxonomic and functional abundance by SOAP2 (Li et al., 2009) with the default parameter.

### Statistical Analyses

Simpson, Shannon, ACE, Chao, and coverage indices were used to compare the α diversity of the bacterial community and they were calculated by Mothur version v.1.30 (http://www.mothur.org/), in R with the VEGAN package. Shannon index based on standardized OTU abundance table was used to evaluate the within-samples diversity (Weiss et al., 2017). Before analysis, all data were normalized by the minimum number of reads per sample. Non-parametric Kruskal-Wallis tests were used to determine the significant differences in α diversity across compartments. The taxonomic dissimilarity analysis between samples was conducted based on the principal coordinate analysis (PCoA) method with Bray-Curtis distances (β diversity) (Lozupone et al., 2011). Variation partitioning analysis (VPA) analysis with two-way PERMANOVA was performed by using VEGAN packages in R software based on OTU relative abundance table. The linear discriminant analysis (LDA) effect size (LEfSe) was used to identify differentially abundant taxa using the non-parametric factorial Kruskal-Wallis H test, A significance alpha of 0.05 and an effect size threshold of 4 were used for all of the biomarkers evaluated.

## Results

### Characterization of Soil Properties

Soil pH significantly increased after lime application (Table 1), from 4.32 to 5.2 and 5.9, respectively, when the different slaked lime was applied to orchard soil. Soil AN content showed a generally declining tendency after lime application (Table 1). SOC, AP, AK, and exchangeable magnesium contents exhibited little variation among treatments (Table 1). In addition, soil pH was negatively correlated with exchangeable aluminum and positively correlated with exchangeable calcium. These results indicated that some soil properties might be closely correlated with the severity of soil acidification of citrus orchards.

**Table 1:** Changes in soil properties of HLB-infected citrus root-zone soil with low slaked lime (SLL) and high slaked lime (SHL) applied to the soil surface.

**Soil parameters**	**SCK**	**SLL**	**SHL**
pH	4.23 ± 0.04a	5.20 ± 0.07b	5.90 ± 0.07c
SOC (g/kg)	11.64 ± 0.92a	11.28 ± 1.82a	12.39 ± 2.01a
AP (mg/kg)	20.49 ± 3.19a	20.82 ± 1.60a	23.92 ± 1.69a
AK (mg/kg)	140.47 ± 8.16a	133.08 ± 11.08a	123.00 ± 4.01a
AN (mg/kg)	104.91 ± 3.03c	90.59 ± 5.16b	70.42 ± 1.12a
Ca^2+^(mg/kg)	705.74 ± 52.15a	1,409.07 ± 86.18b	1,862.6 ± 76.48c
Mg^2+^(mg/kg)	93.85 ± 2.48a	101.12 ± 3.87a	98.29 ± 5.05a
Al^3+^(mg/kg)	240.46 ± 15.53c	146.32 ±15.36b	84.34 ± 9.91a

*Different letters denote significant differences between treatments from a Tukey's HSD test (P < 0.05)*.

*SCK, without lime application*.

### Populations of CLas in Roots and Root Activity

The presence of CLas in roots from different treatments is shown in Table 2. In general, soil amendments reduced the levels of CLas genomes in roots. Compared with the control, the populations of CLas in SLL and SHL treatments significantly decreased by more than 50%, respectively. These results were consistent with those of observation results of HLB symptoms in citrus roots. The severity of HLB disease has been greatly alleviated after lime application, which shows that the difference between fibrous root and biomass could be visually distinguished. Meanwhile, the activity of citrus roots from different types of soils was examined, which was significantly enhanced in the limed soils compared with that in the un-limed soils (Table 2), but there were no significant differences between the SLL and SHL treatments.

**Table 2:** Changes in CLas population and root activity of HLB-infected citrus with low slaked lime (SLL) and high slaked lime (SHL) applied to the soil surface.

**Treatments**	**CLas population (gene copies in 40 mg of fibrous root DNA)**	**Root activity** ** (μg·g^**−1**^ h^**−1**^)**
SCK	(4.37 ± 1.34) × 10^5^	57.89 ± 9.01a
SLL	(9.04 ± 2.34) × 10^4^	98.45 ± 12.32b
SHL	(2.04 ± 1.28) × 10^5^	81.34 ± 10.71b

*Different letters denote significant differences between treatments from a Tukey's HSD test (P < 0.05)*.

*SCK, without lime application*.

### Effects of Lime Application on Taxonomic Features of Root-Endophyte Bacterial Community

The 16S rRNA gene sequencing was used to detect the responses of the endophytic bacterial community resident inside roots of HLB-infected citrus to three soil systems with different soil acidity. After quality filtering, a total of 216,348 high-quality reads were obtained and clustered into 1,305 OTUs for community analysis (Supplementary Table S2). Good's coverage estimators were all above 98.6%, suggesting that the sequencing depths were adequate for the bacterial communities. The number of OTUs and richness indices (Chao1, ACE, and Shannon) in bacterial communities displayed a significant increase in the SLL and SHL samples compared to the SCK samples (Supplementary Table S1). Regarding the Simpson index, SCK showed a significantly higher Simpson index than SLL and SHL (*p* < 0.05). The application of limestone had a significant effect on the α diversity of the HLB-infected root microbial community. However, the difference was not significant between bacterial community diversity indices of the RLL and RHL samples (Supplementary Table S1).

To determine whether the variations of endophytic bacterial community structure in HLB-infected citrus roots are related to soil acidity, we profiled the overall structural changes in bacterial communities in each treatment using Bray-Curtis distances-based PCoA (β diversity) with OTUs annotated at the genus level. The structure of bacterial communities exhibited more similarity in the SLL and SHL treatments, whereas SCK samples form a distinct cluster that was significantly separated from SLL and SHL samples (Supplementary Figure S1). Moreover, the ANOSIM result showed that the differences between groups were greater than those within groups (*R* = 0.88, *p* = 0.001). These results indicated that different rates of limestone application affected the structure of the original bacterial community in HLB-infected citrus roots from highly acidified soils.

**Figure 1:**
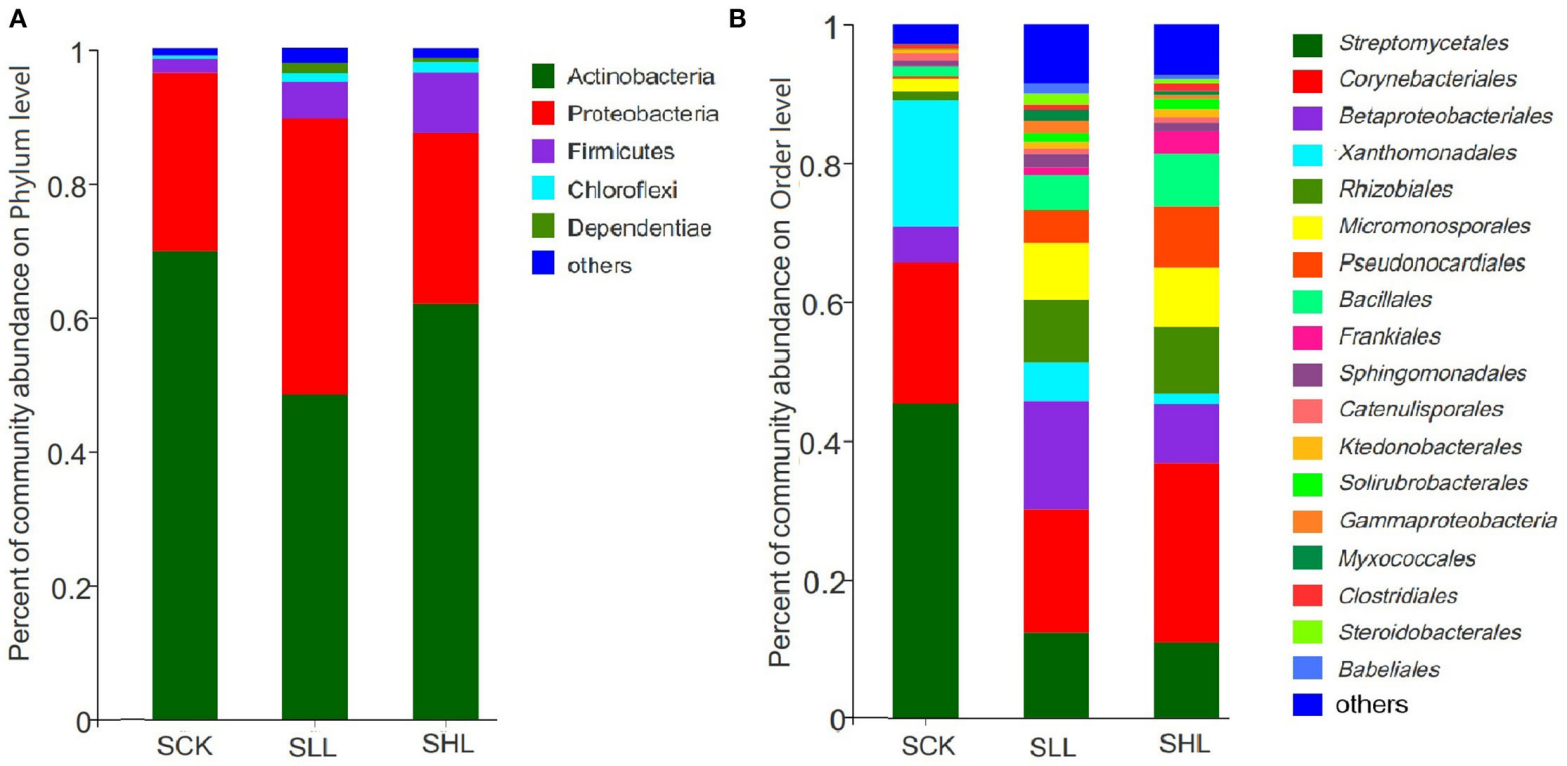
Taxonomic distribution at the phylum **(A)** and Order **(B)** level in HLB-infected citrus root samples based on 16S amplicon. SCK, soil without lime treatment; SLL, soil treated with low-level lime; SHL, treated with high-level lime. Taxa with <1% of reads were combined together as “others”.

Based on the diversity analysis of the bacterial community, qualified sequences were assigned to 26 phyla, 51 classes, 147 orders, 280 families, and 869 genera across all samples. At the phylum level, Actinobacteria (56.17–62.89%), Proteobacteria (27.85–34.26%), Firmicutes (3.32–8.14%), Chloroflexi (0.06–0.11%) and Dependentiae (0.05–0.11%) were top 5 dominant bacteria and the composition was similar in all treatments, accounting for over 97.25% of the total OTUs (Figure 1A). However, Firmicutes and Chloroflexi were relatively more abundant in RLL and RHL roots than in the control, and the relative abundance of Actinobacteria in RCK was higher than that in SLL and SHL. Whereas, no significant difference was detected among the SLL and SHL treatments.

**Figure 2:**
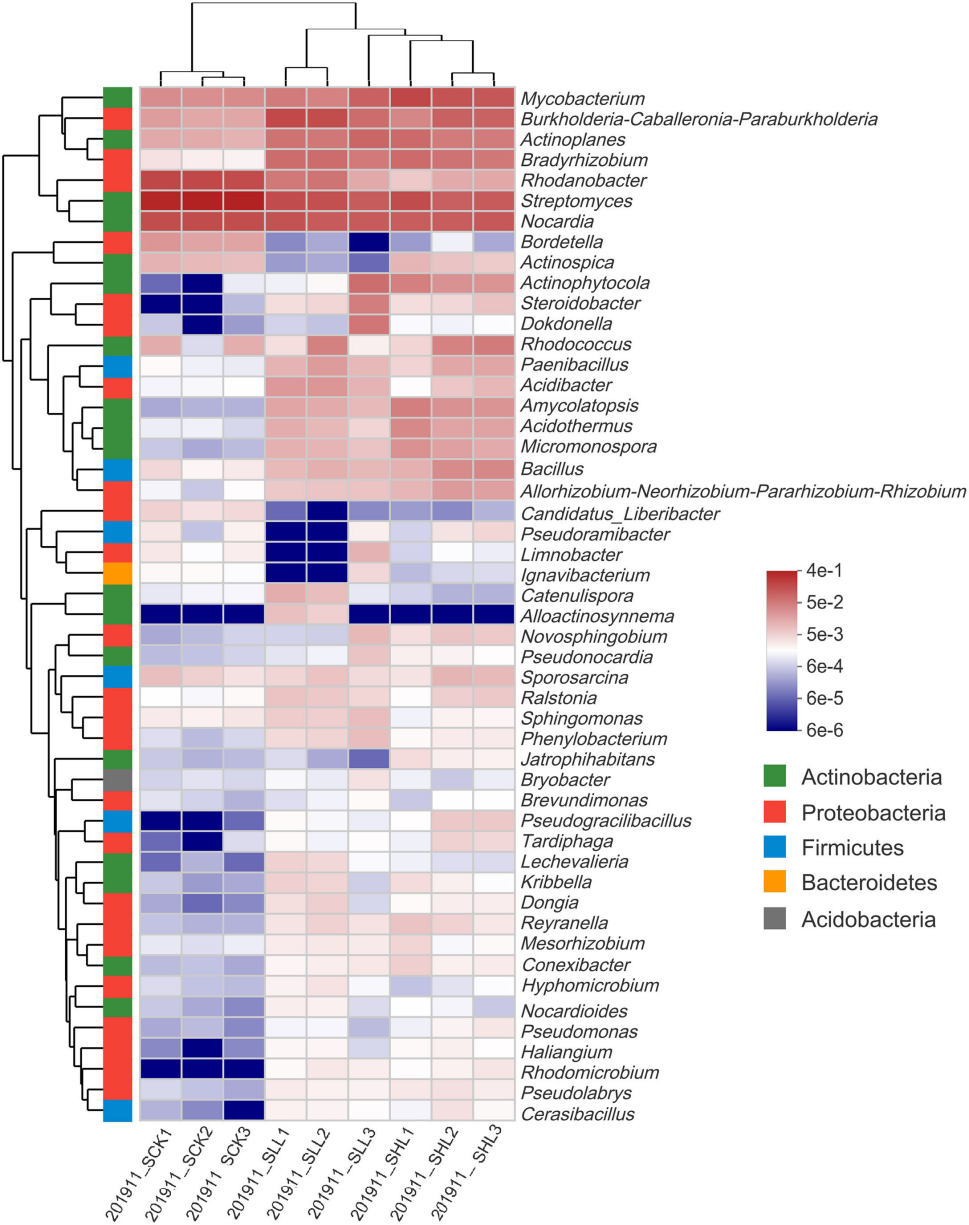
Heatmap for the relative abundance of the 50 most abundant core genera in HLB-infected citrus root samples based on 16S amplicon sequencing. SCK, soil without lime treatment; SLL, soil treated with low-level lime; SHL, treated with high-level lime.

At the order level, such as *Streptomycetales* (45.36%), *Catenulisporales* (1.12%) of *Actinobacteria*, and *Xanthomonadales* (18.16%) of *Gammaproteobacteria*, were present at a higher relative abundance in RCK, whereas multiple orders, such as *Rhizobiales* (9.07–9.49%) of *Alphaproteobacteria, Pseudonocardiales* (4.93–8.56%) of *Actinobacteria*, and *Bacillales* (4.67–7.71%) of *Firmicutes*, were significantly enriched in SLL and SHL (Figure 1B). At lower (genus and OTUs) taxonomic ranks, we first selected the genera with the top 50 average relative abundance for heatmap analysis (Figure 2). The result showed that *Streptomyces* (45.38%), *Nocardia* (16.12%), and *Rhodanobacter* (17.82%) were the predominant bacterial genera in RCK, which gradually decreased in the limestone application group. As expected, the relative abundance of *Candidatus Liberibacter* in RCK was much higher than that in RLL and RHL, which was consistent with the changing trend of absolute abundance of HLB pathogen root samples determined by qPCR. On the other hand, most other genera, such as *Bacillus, Bradyrhizobium, Paenibacillus, Pseudomonas*, and *Burkholderia-Caballeronia-Paraburkholderia*, are generally considered to contain many plant-beneficial bacteria, were more abundant in SLL and SHL compared to SCK (Figure 2).

**Figure 3:**
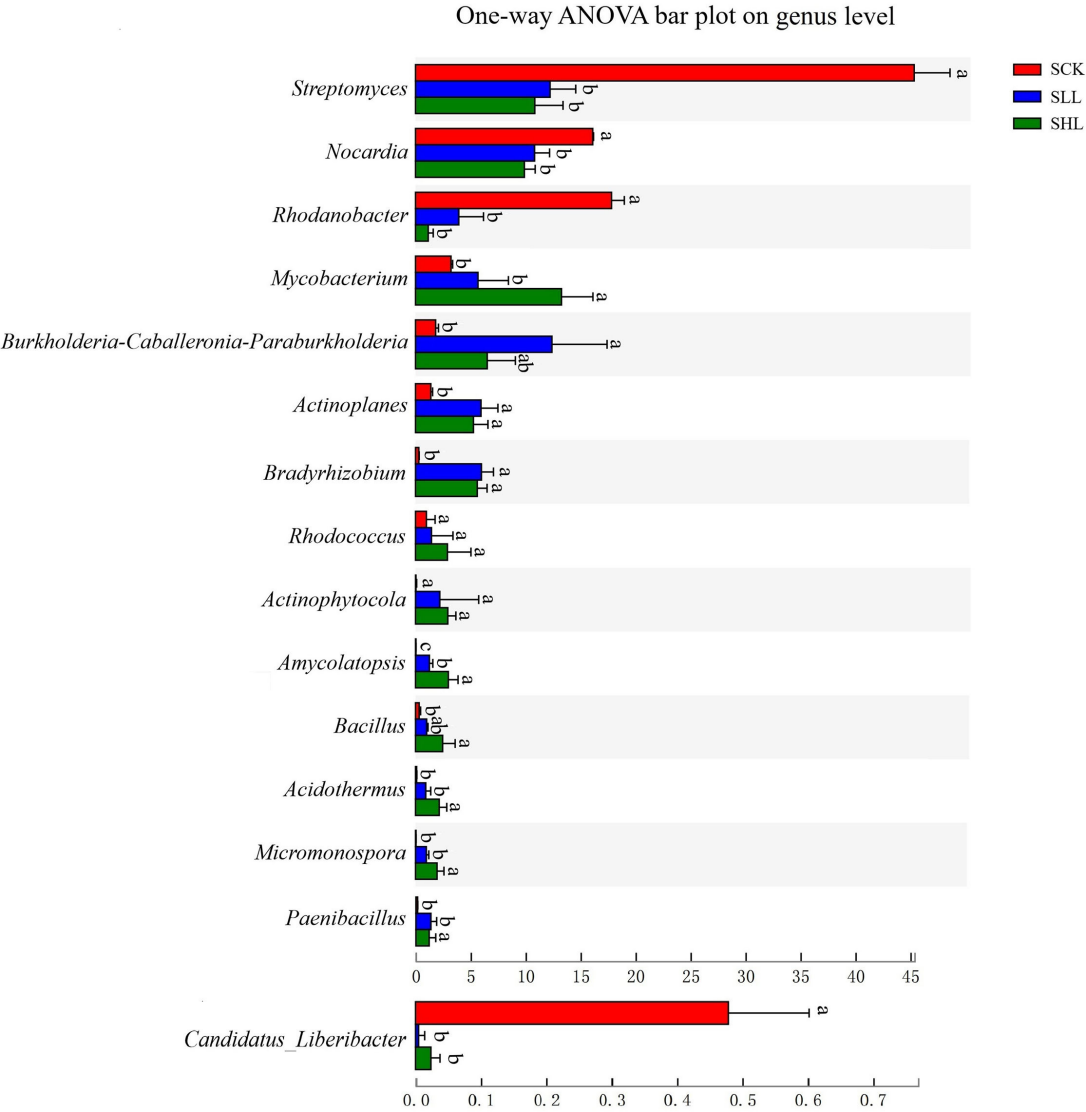
Relative abundances of bacterial taxa at the genus level showed significant differences among HLB-infected root samples from SCK, SLL, and SHL soils. SCK, soil without lime treatment; SLL, soil treated with low-level lime; SHL, treated with high-level lime. Differences were considered significant at *p* < 0.05 by the Kruskal Wallis H test and respective significant differences were marked using different letters among indicated groups.

Also, Kruskal Wallis H test results (*p* < 0.05) indicated that 13 of the top 15 dominant bacteria from SCK at genus level and *C. Liberibacter* had significant abundant differences in response to different soil acidity (Figure 3). Subsequently, the LEfSe results used to examine taxa differences from phylum to genus between the treatment (SLL or SHL) and control groups (SCK) were represented in a multi-level species hierarchy tree diagram (Supplementary Figure S2) and a linear discriminant analysis table (Table 3) (logarithmic LDA score > 4). In the SCK sample, 8 bacterial taxa (Group 1) belong to two ordres (*Streptomycetales* and *Xanthomonadales*), three families (*Nocardiaceae, Streptomycetaceae*, and *Rhodanobacteraceae*) and three genera (*Nocardia, Streptomyces*, and *Rhodanobacter*) were significantly more abundant in pairwise comparisons of treatments. For samples from amended soils, SLL and SHL treatments shared 16 core bacterial taxa (Group 2) taken as potential taxon indicators for response to soil acidity of HLB-infected roots. Among those taxa, phylum Firmicutes had significantly higher relative abundance in SLL and SHL treatments. At the genus level, SHL treatment exclusively and significantly increased the relative abundance of 5 additional genera (Group 4), including *Bacillus, Acidothermus, Micromonospora, Amycolatopsis*, and so on.

**Table 3:** Taxonomic biomarkers of bacterial communities in response to the lime application (*p* < 0.05, logarithmic LDA logarithmic score ≥ 4).

**Taxonomic biomarkers**					**LDA value (log10)**	
**Phylum**	**Class**	**Order**	**Family**	**Genus**	**SCK and SLL**	**SCK and SLL**
Actinobacteria	*Actinobacteria*	*Corynebacteriales*	*Nocardiaceae*		4.34	4.36
Actinobacteria	*Actinobacteria*	*Corynebacteriales*	*Nocardiaceae*	*Nocardia*	4.35	4.51
Actinobacteria	*Actinobacteria*	*Streptomycetales*			5.22	5.23
Actinobacteria	*Actinobacteria*	*Streptomycetales*	*Streptomycetaceae*		5.18	5.21
Actinobacteria	*Actinobacteria*	*Streptomycetales*	*Streptomycetaceae*	*Streptomyces*	5.23	5.18
Actinobacteria	*Gammaproteobacteria*	*Xanthomonadales*			4.84	4.93
Actinobacteria	*Gammaproteobacteria*	*Xanthomonadales*	*Rhodanobacteraceae*		4.78	4.91
Actinobacteria	*Gammaproteobacteria*	*Xanthomonadales*	*Rhodanobacteraceae*	*Rhodanobacter*	4.83	4.92
Actinobacteria	*Actinobacteria*	*Corynebacteriales*	*Mycobacteriaceae*		4.17	4.71
Actinobacteria	*Actinobacteria*	*Corynebacteriales*	*Mycobacteriaceae*	*Mycobacterium*	4.14	4.73
Actinobacteria	*Actinobacteria*	*Micromonosporales*			4.52	4.55
Actinobacteria	*Actinobacteria*	*Micromonosporales*	*Micromonosporaceae*		4.52	4.54
Actinobacteria	*Actinobacteria*	*Micromonosporales*	*Micromonosporaceae*	*Actinoplanes*	4.37	4.33
Actinobacteria	*Actinobacteria*	*Pseudonocardiales*			4.34	4.63
Actinobacteria	*Actinobacteria*	*Pseudonocardiales*	*Pseudonocardiaceae*		4.38	4.63
Actinobacteria	*Actinobacteria*	*Pseudonocardiales*	*Pseudonocardiaceae*	*Actinophytocola*	4.08	4.18
Firmicutes					4.20	4.52
Firmicutes	*Bacilli*				4.18	4.49
Firmicutes	*Bacilli*	*Bacillales*			4.14	4.46
Proteobacteria	*Alphaproteobacteria*				4.74	4.72
Proteobacteria	*Alphaproteobacteria*	*Rhizobiales*			4.56	4.60
Proteobacteria	*Alphaproteobacteria*	*Rhizobiales*	*Xanthobacteraceae*		4.52	4.51
Proteobacteria	*Alphaproteobacteria*	*Rhizobiales*	*Xanthobacteraceae*	*Bradyrhizobium*	4.47	4.43
Proteobacteria	*Gammaproteobacteria*	*Betaproteobacteriales*	*Burkholderiaceae*	*Burkholderia-Caballeronia-Paraburkholderia*	4.71	4.33
Proteobacteria					4.89	
Proteobacteria	*Gammaproteobacteria*	*Betaproteobacteriales*			4.71	
Proteobacteria	*Gammaproteobacteria*	*Betaproteobacteriales*	*Burkholderiaceae*		4.70	
Firmicutes	*Bacilli*	*Bacillales*	*Bacillaceae*	*Bacillus*		4.02
Actinobacteria	*Actinobacteria*	*Frankiales*	*Acidothermaceae*	*Acidothermus*		4.01
Actinobacteria	*Actinobacteria*	*Micromonosporales*	*Micromonosporaceae*	*Micromonospora*		4.01
Actinobacteria	*Actinobacteria*	*Pseudonocardiales*	*Pseudonocardiaceae*	*Amycolatopsis*		4.16
Actinobacteria	*Actinobacteria*	*Pseudonocardiales*	*Pseudonocardiaceae*	*unclassified_Pseudonocardiaceae*		4.03

*SLL, soil treated with low-level lime; SHL, treated with high-level lime*.

### Effects of Lime Application on Taxonomic Features of Rhizoplane Microbial Community

We collected the rhizoplane soil samples from HLB-diseased citrus trees under different soil acidity for metagenomic sequencing analysis. More than 420.6 million clean reads were generated for the 9 samples, yielding a total of 3,139,960 contigs after assembly. The metagenomic sequences were chosen to define the change of core rhizoplane microbiome from highly acidified to lime-amended soils duo this method could provide more comprehensive taxonomic information given the community compositions. A total of 12,358 species were obtained after taxonomic annotations, 90.37% of which were prokaryotic (bacteria and archaea), and a small fraction of species were annotated as eukaryotes (including fungi, protozoa, algae, and plants) and viruses (Supplementary Table S2).

**Figure 4:**
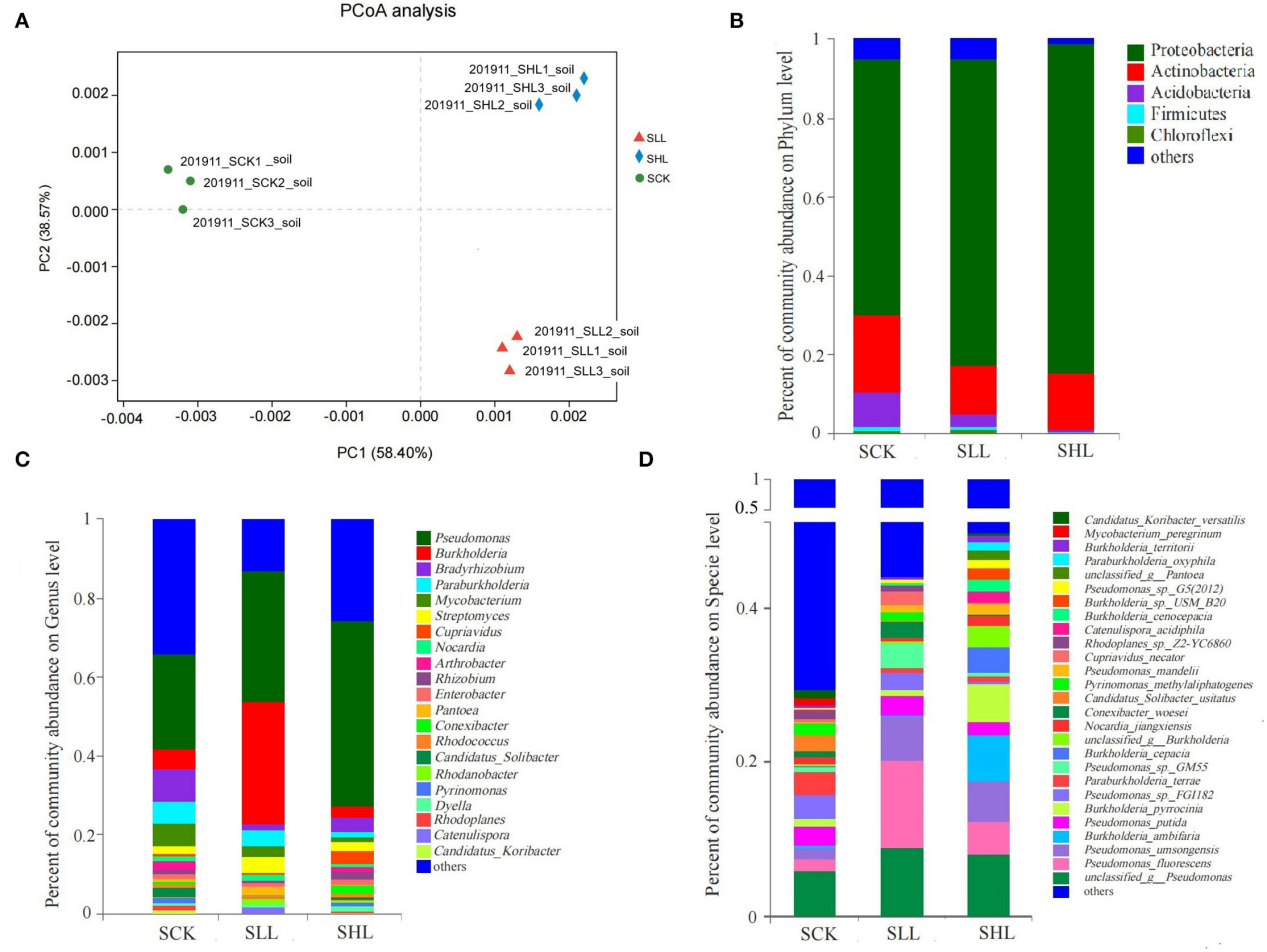
Taxonomic composition of HLB-infected citrus rhizoplane microbiome. **(A)** Principal-coordinate analysis (PCoA) based on bray_curtis distances for the rhizoplane microbiome in acidified (SCK) or amended soils (SLL and SHL) at the specie level. Taxonomic distribution at the Phylum **(B)**, Genus **(C)**, and Specie **(D)** level in HLB-infected citrus rhizoplane soil samples. SCK, soil without lime treatment; SLL, soil treated with low-level lime; SHL, treated with high-level lime. Taxa with <1% of reads were combined together as “others”.

We then investigated the taxonomic distinctiveness of the HLB-infected citrus rhizoplane microbiome under different soil acidity. No significant difference in α diversity between the treatments at the species level was seen, suggesting that limestone application alphas did not significantly alter the overall richness (represented by the Chao index) and diversity (represented by the Shannon index) of the root-associated microbiome. However, PCoA based on Bray_Curtis distance (β diversity) revealed that the community composition of the rhizoplane at the species level in SLL and SHL differed from that in SCK (Figure 4A). We compared the relative abundances of microbial communities in all the samples at both high (phylum) and low (genus) taxonomic ranks to identify those community members differing in abundance in citrus rhizoplane soil from acidified soils and amended soils (Figures 4B–D). The dominant prokaryotic phyla found in the HLB-infected citrus rhizoplane from different soil types, including Proteobacteria, Actinobacteria, Acidobacteria, and Firmicutes. The results revealed that the relative abundance of Proteobacteria was significantly enriched with the application of limestone, whereas multiple bacterial phyla, such as Actinobacteria, Acidobacteria, and Firmicutes of HLB-infected citrus rhizoplanic microbes, were depleted in amended soils than that in acidified soils (Figure 4B). A more detailed multiple comparisons at the genus level between the SCK, SLL, and SHL microbial communities were performed. *Pseudomonas, Bradyrhizobium*, and *Burkholderia* were ranked as the top three dominant genera in all samples. Among the 21 genera (relative abundance ≥ 1%) identified in all samples, 3 genera *Pseudomonas, Streptomyces*, and *Dyella*, exhibited significantly increased relative abundance in the rhizoplane soil with the increased application rate of limestone, while the relative abundance of 9 genera showed significantly depleted in SLL and SHL compared in SCK, including *Bradyrhizobium, Paraburkholderia, Mycobacterium, Arthrobacter*, and *Candidatus Solibacter*. The change in relative abundance of the other 7 genera is contradictory under different soil acidity conditions, for example, the relative abundance of *Burkholderia*, a key taxon in the root microbiome of healthy citrus (Zhang et al., 2017), decreased slightly in SLL and increased significantly relative abundance in SHL (*p* < 0.05) compared to SCK (Figure 4C). Specifically, five of the top 10 species with the relative abundance were significantly higher in the amended soils than in the control soils, including unclassified_g__Pseudomonas, *Pseudomonas fluorescens, Pseudomonas umsongensis, Burkholderia ambifaria*, and *Pseudomonas sp*. GM55. Meanwhile, the other two species *Burkholderia pyrrocinia* and *Burkholderia cepacia* are significantly enriched in SLL than SCK, while it is lower in SHL (Figure 4D).

In addition, RDA was performed to elucidate relationship among environmental factors and microbial community structure at genus level (**Figure 6A**). Among these soil environmental factors, significant influences of pH (r^2^ = 0.975, *p*-value = 0.002), AN (r^2^ = 0.901, *p*-value = 0.001), Ca2+ (r^2^ = 0.962, *p*-value = 0.002), and Al3+ (r^2^ = 0.943, *p*-value = 0.001) were observed in microbial community structure, indicating that soil pH, exchangeable cations (Ca^2+^ and Al^3+^) and AN were significant factors regulating the microbial composition. In particular, the relative abundance of *Pseudomonas* and *Burkholderia* was positively correlated with soil pH in SLL and SHL samples.

### Effects of Lime Application on Functional Features of Rhizoplane Microbial Community

**Figure 5:**
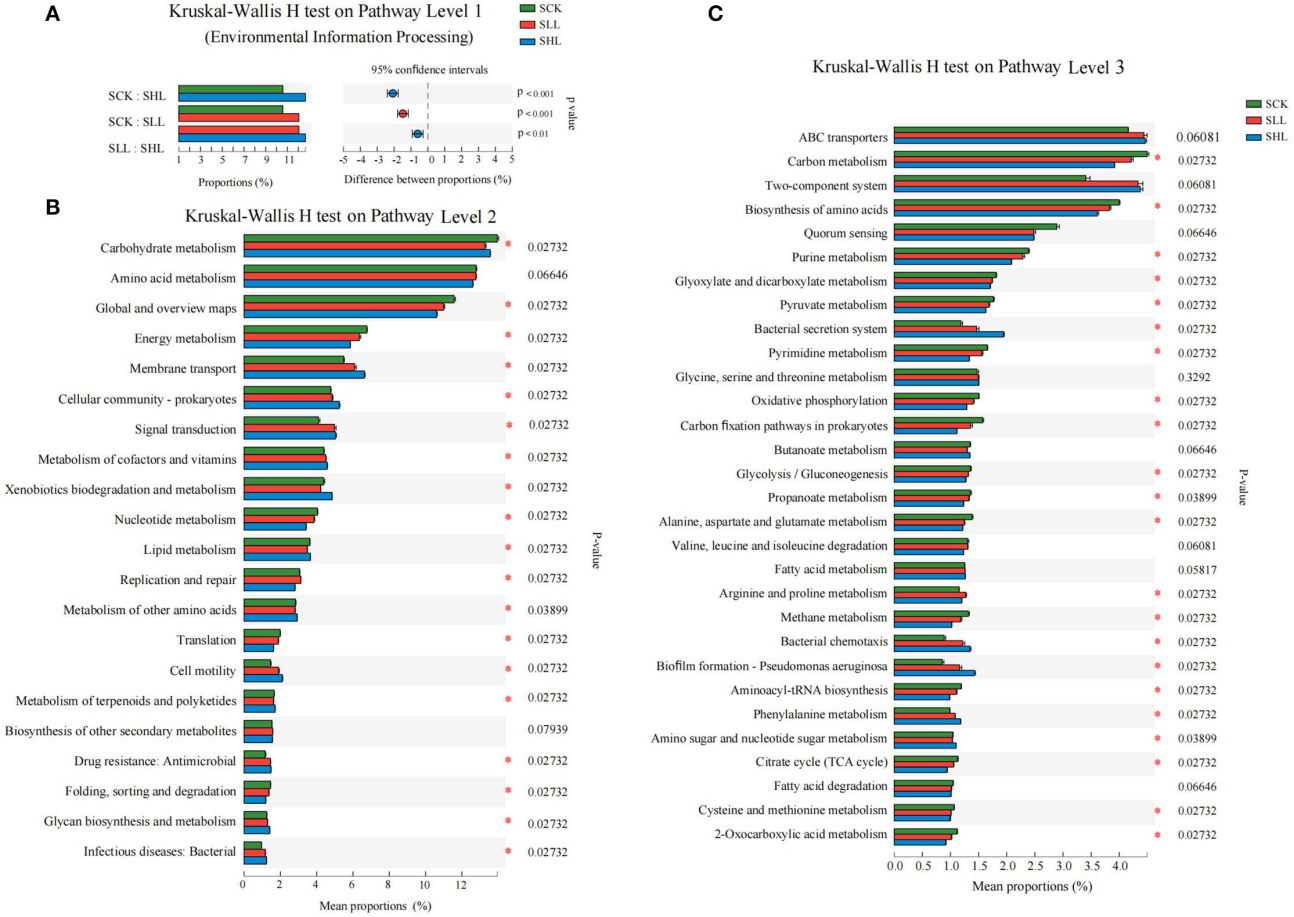
Relative abundance of predicted genes related to KEGG pathways at levels 1 **(A)**, 2 **(B)**, and 3 **(C)** showed significant differences among HLB-infected citrus rhizoplane soil samples. SCK, soil without lime treatment; SLL, soil treated with low-level lime; SHL, treated with high-level lime. Differences were considered significant at *p* < 0.05 by the Kruskal Wallis H test.

The metagenomic sequences also provided functional information about a given taxon. Therefore, apart from phylogenic insights, the metagenomic analysis also provided an opportunity to assess the functional potentials associated with the soil microbial community. Functional annotation was performed for non-redundant genes by blasting against the KEGG Orthology (KO). A total of 7,712 KOs were identified from all metagenomes, and they were mainly involved in 6, 45, and 399 KEGG pathways at three levels, respectively. The majority of sequences were functionally associated with metabolism (66.72–68.77%), environmental information processing (9.58–11.66%), cellular processes (7.58–8.35%), genetic information processing (5.83–6.82%), human diseases (4.57–5.27%), and organismal systems (2.17–2.67%) at KEGG level 1 pathways. The results of the Kruskal Wallis H test indicated that the relative abundance of predicted genes involved in environmental information processing was significantly higher in amended soil samples (SLL and SHL) than in the acidified soil sample (SCK) (Figure 5A).

Furthermore, we also investigated the differences of 21 and 30 dominant categories (relative abundance > 1% of the total observed pathways) at KEGG levels 2 and 3 pathways between the treatments and control samples, respectively. Application of limestone significantly enriched the relative abundance of membrane transport, cellular community-prokaryotes, signal transduction, metabolism of cofactors and vitamins, xenobiotics biodegradation and metabolism, cell motility, antimicrobial drug resistance, glycan biosynthesis, and metabolism, and infectious bacterial diseases in SLL and SCK than that in SCK samples (all *p* < 0.05), while the rest 10 categories, except for the KEGG pathways amino acid metabolism and biosynthesis of other secondary metabolites, were depleted in SLL and SHL compared with SCK (Figure 5B) at level 2 pathways. Among KEGG level 3 pathways, the relative abundance of the ABC transporters, two-component system, bacterial secretion system, arginine and proline metabolism, bacterial chemotaxis, biofilm formation, flagellar assembly, and phenylalanine metabolism, which belong to environmental information processing and cellular processes, increased after limestone application, and these functions are very critical for microorganisms to adapt to the plant rhizoplane environment (Figure 5C).

**Figure 6:**
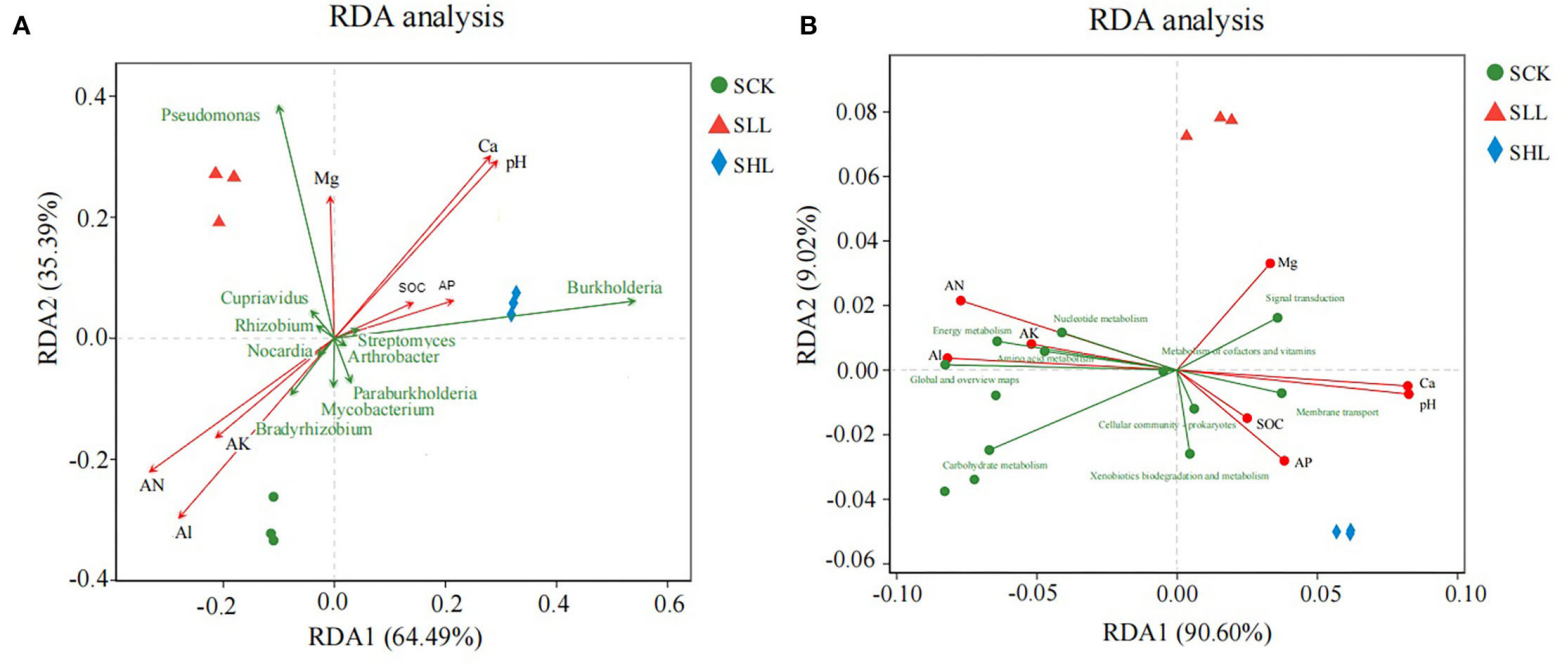
Redundancy analysis plots of the correlation between the structure **(A)** and function **(B)** of the microbial community in HLB-infected citrus rhizoplane and environmental factors during the neutralization of soil acidity.

The relationship between environmental parameters and microbial community functional properties (Figure 6B) was further confirmed by PCA based on the abundance of the KEGG (level 2) module. There are four soil environmental parameters that have significant effects on the functional pathways of rhizoplane microbiome, including pH (r^2^ = 0.979, *p*-value = 0.002), AN (r^2^ = 0.892, *p*-value = 0.002), Ca^2+^ (r^2^ = 0.965, *p*-value = 0.002), and Al^3+^ (r^2^ = 0.935, *p*-value = 0.001) (Figure 6B). Especially, it is found that there is a strong positive correlation between soil pH and several functional pathways, such as signal transduction, membrane transport, cellular community and Xenobiotics biodegradation, and metabolism in amended soil samples.

### Changes in Nitrogen and Sulfur Metabolism With Neutralization of Soil Acidity

**Figure 7:**
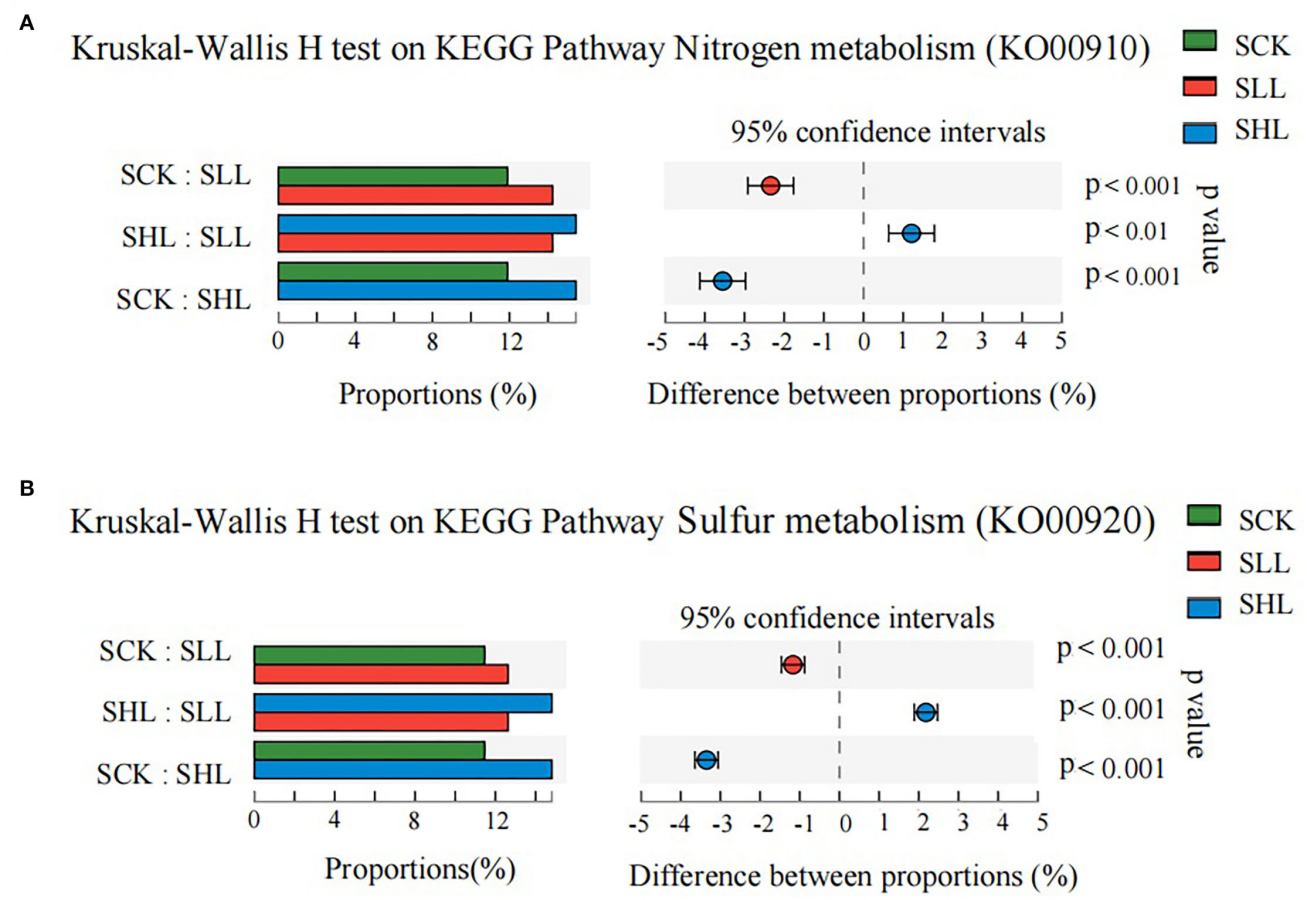
The relative abundance of predicted genes related to nitrogen metabolism (KO00910) **(A)** and sulfur metabolism (KO00920) **(B)** showed significant differences among HLB-infected citrus rhizoplane soil samples. SCK, soil without lime treatment; SLL, soil treated with low-level lime; SHL, treated with high-level lime. Differences were considered significant at *p* < 0.05 by the Kruskal Wallis H test.

In particular, the functional categories nitrogen metabolism (KO00910) and sulfur metabolism (KO00920) were significantly enriched from acidified soils to improved soils (Figure 7). The results revealed that a variety of enzymes encoded by corresponding functional genes were involved in nitrogen and sulfur cycling and influenced by soil acidity in HLB-infected citrus rhizoplane soil (Supplementary Figure S3A). In the nitrogen cycle, the nitrite reductase (*nirB* and *nirD*) and the nitrate reductase (*narG, narH, and narI*) are involved in dissimilatory nitrate reduction to ammonium (DNRA) and were more abundant in the SLL and SHL groups. While ferredoxin-nitrite reductase (*nirA*) and glutamate synthase (*GLT1*) were more abundant in the SCK groups. Moreover, two genes *arcC* and *hcp* encoding carbamate kinase and hydroxylamine reductase, respectively, were generally increased in abundance from SCK to SHL. In the sulfur cycle, the assimilation sulfate reduction pathway is important to incorporate sulfide into organic compounds. In this study, a majority of these genes that participated in the assimilatory sulfate reduction pathway were more abundant in the SLL and SHL groups rather than in the SCK (Supplementary Figure S3B), such as adenylyl-sulfate kinase (*cysC*) and assimilatory sulfite reductase (*cysI* and *cysJ*). Only assimilatory sulfite reductase (*sir*) showed a slight decline. Meanwhile, the relative abundance of genes *cysE, metA*, and *metB* related to cysteine and methionine metabolism decreased after the application of limestone. Our findings indicated that the relative abundance of genes related to nitrogen and sulfur cycling presented diverse and intricate changes during the soil improvement because, besides soil acidity, there are many other soil physicochemical factors that can cooperatively influence the microbe-driven metabolism cycle to regulate the availability and quantity of different forms of nitrogen and sulfur in soils.

### Linking the Rhizoplane Taxonomic and Functional Properties to Different Soil Types

**Figure 8:**
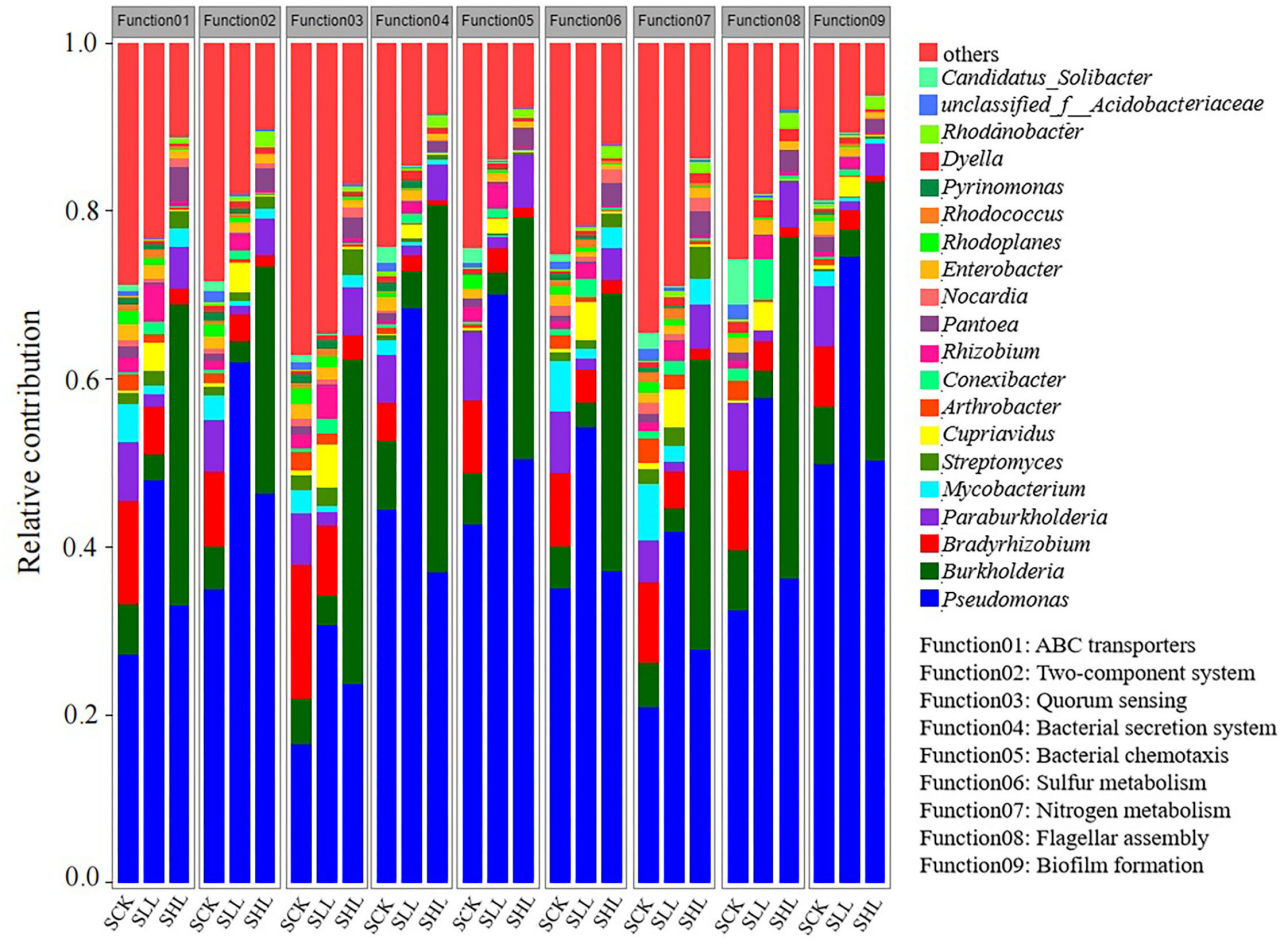
The relative contribution of different taxa (the genus with more than 1% relative abundance) to identified rhizosphere functional attributes in different soil samples. SCK, soil without lime treatment; SLL, soil treated with low-level lime; SHL, treated with high-level lime.

To visualize the association between rhizoplane taxonomic and functional properties during the acid soil improvement, we determined the taxonomic origin of rhizoplane-enriched functional attributes for all samples. The contribution of the top 20 genera in relative abundance to nine clustered categories enriched KOs at KEGG pathway level 3 were chosen and analyzed (Figure 8). These functional categories involved in soil nutrient status cycling, such as nitrogen and sulfur metabolism, they also related to host-microbe interactions, including ABC transporters, two-component system, bacterial secretion system, quorum sensing, biofilm formation, bacterial chemotaxis, and flagellar assembly. The genera *Pseudomonas* and *Streptomyces* was the main contributor to these functions and contributed significantly more to the SLL and SHL samples (paired *t*-test for each selected functional category, all *p* < 0.01). Especially, the normalized total relative contribution of *Pseudomonas* for the nine functional categories was 43.01 ± 1.59% and 31.66 ± 0.46% for SLL and SHL samples, respectively, while was only 21.98 ± 1.12% for SCK samples. A reduced contribution of *Bradyrhizobium* and *Mycobacterium* for the SLL and SHL samples was also observed (two taxa together accounting for 14.17 ± 0.94% of the normalized total relative contribution for the SCK samples and 5.52 ± 0.4%, 4.28 ± 0.24% for SLL and SHL samples, respectively). The relative contribution of *Burkholderia* to these functional categories for the SLL and SHL samples ranged from 2.39 to 4.44% and 27.04 to 43.67%, respectively. Compared with SCK samples, the relative contribution of *Burkholderia* to the SHL sample significantly increased but decreased in SLL samples (paired *t*-test, all *p* < 005).

## Discussion

In this study, the remediation effects of limestone application on root physiological traits, soil physiochemical properties, and microbial community structure and function in highly acidified and HLB-infected citrus orchards were investigated. Our data demonstrated that the application of slacked lime improved the root activities of HLB-infected citrus, a finding that is with previous studies on ryegrass (Du et al., 2021), wheat (Haling et al., 2010), clovers (Brauer et al., 2002), Sitka spruce (Kakei and Clifford, 2002) and citrus (Li et al., 2020), and reduced the population of HLB pathogen in the roots. In addition, due to the red soil of subtropical China has inherent fragile properties, including low pH, low organic matter and low fertility, the overall soil quality of citrus orchards in south China belongs to the lower middle level according to the SQI grades (Cheng et al., 2016). These environmental factors can have a positive correlation with the infection risk, propagation, and development of plant diseases caused by poor plant vigor (Larkin, 2015). Previous studies have shown the application of limestone can improve soil nutrient status, reduce soil acidification and neutralize toxic aluminum in both annual and perennial planting systems (Natale et al., 2012; Corrêa et al., 2018; Li et al., 2019; Lauricella et al., 2021). Combined with our research results, we believe that amending soil acidification can help optimize the soil nutrient status of citrus orchards and mitigate HLB disease progress.

Plant root loss caused by pathogen infestation and extreme soil environment could limit the water and nutrient uptake of trees, making them more vulnerable to biotic and abiotic stress (Ghimire et al., 2020). The root is the earliest and most common site for CLas colonization and proliferation, a significant damage to feeder roots has occurred on presymptomatic HLB-affected trees (Johnson et al., 2014), which means that CLas infestation could directly cause the loss of roots rather than the carbohydrate starvation caused by phloem plugging of symptomatic HLB affected citrus. The loss of fibrous root function may also cause intolerance to extreme environments and other plant diseases. Recent evidence indicates that the HLB infection was actually accompanied by other citrus pests and diseases under filed conditions (Duan et al., 2021). Prior CLas infection on roots accelerates *Phytophthora* spp. infection and further damage to the fibrous roots, which is due to the fact that the HLB-infected roots are more attractive to swimming zoospores and have lower resistance to root invasion (Graham et al., 2013). In this study, the soil improvement of acidified citrus orchard partially reduced the damage of roots caused by CLas infection. As a result, the CLas pathogen content in the infected roots decreased significantly and the activity of the infected roots increased significantly after soil improvement. Therefore, the restoration of affected fibrous root function by optimizing soil pH, which is an essential cultural practice for sustainable plant root vigor and a fundamental component of plant disease control and tolerance.

Another contributing factor to this tolerance to HLB probably is the intense reshaping of microbial community diversity and structure in citrus roots after soil improvement. A stable correlation between plants and the endophytic microbial community is very important to maintaining the health and productivity of hosts (Blaustein et al., 2017). In this study, the diversity of root bacterial communities in the amended soils is strongly increased compared with the control soils. Previous studies generally believed that the diversity of the citrus endophytic microbiota was negatively associated with HLB disease severity (Trivedi et al., 2010). Meanwhile, the microbiota of roots was significantly disrupted undergoing rapid HLB progression and lacked resilience under pathogen or disease stress (Ginnan et al., 2020). When examining the root (endo-) bacterial communities in this study, the most dominant bacterial phyla Firmicutes and Chloroflexi were at increased levels in trees with higher vigor after soil improvement, but the levels of Actinobacteria were opposite. As the severity of HLB decreases, Ginnan et al. (2020) also confirmed that the relative abundance of phylum Actinobacteria decreased in HLB-infected trees, which was largely represented by *Streptomyces* spp. known as prolific producers of antibiotics. However, this HLB disease-induced enrichment of *Streptomyces* in the roots is not synchronized with the phase of CLas colonization and is insufficient to mitigate HLB symptoms in infected plants (Ginnan et al., 2020). Notably, there was a sharp increase in the relative abundance of some bacterial genera considered beneficial in the roots of diseased trees after soil improvement, which contributed to the vitality of the whole roots and plants and was critical for inducing plant resistance (Compant et al., 2005; Trivedi et al., 2010). Therefore, we speculate that the restoration and bolstering of potentially beneficial bacteria in roots is one of the main contributors to the more vibrant root observed in HLB trees from amended soils.

The infection of CLas not only restricts phloem transportation of photoassimilates and induces root decline (Wang et al., 2017a), but also negatively modifies the structure and functional diversity of microbial communities in the citrus rhizosphere (Trivedi et al., 2012; Zhang et al., 2017). The relative abundances of the main phylum Proteobacteria in infected rhizospheric microbes was nearly five times less compared with that of healthy citrus, while the other dominant bacteria belonging to Actinobacteria, Acidobacteria, and Firmicutes phyla were significantly enriched in HLB-infected samples (Trivedi et al., 2012). As a result, HLB caused the roots to acidify the soil more than non-HLB controls (Ebel et al., 2019), this phenomenon is probably due to the change of microbial community structure in the citrus rhizosphere. And this adverse effect of CLas infection makes citrus more susceptible to other extreme environments and secondary diseases than healthy trees (Aritua et al., 2013; Johnson et al., 2014). A complex plant root-associated microbial community is considered the second genome of the plant because stable and robust soil microbes are essential and play a key role in the availability and circulation of soil nutrients and even can improve stress tolerance or inhibit pathogens (Pang et al., 2021). Therefore, the improvement of acidified soil in citrus orchards where HLB disease is prevalent in this study does not only help to improve the chemical properties of soil, including supplementing and increasing calcium content and alleviating aluminum toxicity in acidic soils, which is beneficial to the growth and vigor of citrus trees but also restore the seriously damaged soil microbial community associated with CLas infection to a certain extent in a pH-driven way. Specifically, the relative abundances of phyla Actinobacteria, Acidobacteria, and Firmicutes, which are positively related to the occurrence of HLB (Trivedi et al., 2012), decreased significantly after soil improvement; while the relative abundance of Proteobacteria increased significantly. Members of phylum Proteobacteria were shown to have disease-suppressive activity governed by non-ribosomal peptide synthetases (Mendes et al., 2011).

Especially, the relative abundance of most of the top 10 dominant species in citrus rhizoplane increased significantly after soil improvement. These species are affiliated with the genus *Pseudomonas* and *Burkholderia*, which were generally considered to contain a variety of beneficial microorganisms associated with promoting plant growth and inducing plant resistance (Ganeshan and Kumar, 2005; Stopnisek et al., 2014). For instance, among these enriched species, *Pseudomonas fluorescens, Burkholderia ambifaria, Burkholderia pyrrocinia*, and *Burkholderia cepaci* are naturally beneficial bacteria with proven biocontrol properties (Ren et al., 2011; Chapalain et al., 2013; Jung et al., 2018; Mullins et al., 2019). Riera et al. (2017) also reported that *Burkholderia metallica* strain A53, *Burkholderia territorii* strain A63, *Pseudomonas granadensis* strain 100, and *Pseudomonas geniculata* strain 95 from healthy-looking citrus rhizosphere in severely HLB-diseased citrus grove showing antibacterial activity against *Agrobacterium tumefaciens* and *Sinorhizobium meliloti* closely related to CLas pathogens. Although the HLB escape plants have the same genotype as the symptomatic trees, the difference in microbial community composition enriched in beneficial traits may result in the promotion of stress tolerance and disease inhibition of escape trees by competing resources or providing plant growth-promoting factors (Riera et al., 2017; Wang et al., 2017b). Furthermore, our research and previous studies have consistently shown that soil pH was the imperative factor influencing community composition, which may be related to the relationship between soil acidity and relative abundances of dominant and core bacteria in diseased citrus root or rhizoplane. Therefore, we speculated that acidic soil improvement can recruit and enrich specific beneficial microbes from root-associated microbiota in a soil pH-driven microbial redistribution pattern, which can promote tolerance to HLB and other stress.

The composition of microbial community and the relative abundance of core bacteria groups in diseased citrus rhizoplane vary with soil acidity, thus, we thought that the functions of rhizoplane microbes might be different between the acidic soils and the amended soils. Beneficial plant-microbe associations modified the metabolic pathways of rhizoplane microbes responding to the external environment, which may play critical roles in plant health and disease resistance. Our results indicated that there is a strong correlation between the environmental factor pH and microbial functions. These functional categories related to host-microbe interactions and soil nutrient status cycling, including two-component system, biofilm formation, bacterial chemotaxis, flagellar assembly, bacterial secretion system, and nitrogen and sulfur metabolism, were obviously enriched in diseased citrus rhizoplane with the neutralization of soil acidity. Firstly, two-component regulatory systems help the rhizobacteria recognize and adapt to environmental changes (Heeb and Haas, 2001). In addition, recent studies highlighted the importance of biofilm formation in initiating and maintaining contact with the host, which enables bacterial populations adhere to plant surfaces, thus affecting the colonization tendency of beneficial bacteria (Ramey et al., 2004; Farrar et al., 2014). Meanwhile, bacterial chemotaxis provides a competitive advantage for motile flagellated bacteria, especially beneficial plant-associated bacteria, in response to root exudates and colonization of plant root surfaces (Scharf et al., 2016). The above functions are the intrinsic components of plant-microbe interaction and are also the prerequisites for establishing stable and beneficial associations. After successful colonization of plant roots, the effectiveness of beneficial rhizobacteria usually depends on the secretion system to help the host to obtain nutrition from the soil, improve plant adaptability, and inhibit pathogen colonization (Lucke et al., 2020). These viewpoints combined with our findings suggested that acidic soil improvement could increase the vitality and motility in soil microbial communities and assist HLB-infected roots to recruit beneficial soil microbes, which play an important role in the secretion of antibiotics and the activation of the plant immune system.

In addition, soil microbes can utilize various forms of inorganic nitrogen to synthesize the organic biomass, which plays an important role in the soil's total nitrogen cycle (Thus, 2005). DNRA consumes nitrogen oxide pool and preserves bioavailable nitrogen in the soil system, which produces soluble ammonium rather than unreactive N_2_ or N_2_O (Marchant et al., 2014). The enhanced DNRA of soil microbes in the amended soils might promote the recycling of nitrogen, which is very important for the level of soil fertility. However, not all nitrogen metabolism is related to the synthesis of biomass in organic form, because some bacteria need to utilize the energy released by nitrogen reactions to maintain their life activities (Thus, 2005). As in the case of nitrogen, sulfur is required by bacteria as an essential nutrient for cellular biosynthesis and the transformation of sulfur in the environment mainly depends on the activities of microorganisms (Klotz et al., 2011). Normally, most microorganisms acquire sulfur and synthesize the organic sulfur compounds through assimilatory sulfate reduction (Kushkevych et al., 2020). In this process, sulfate is reduced to hydrogen sulfide and then participates in sulfur-containing amino acids biosynthesis. In this study, the abundance of most genes (*cysC, cysI*, and *cysJ*) related to the sulfate reduction pathway was increased, meanwhile, the abundance of genes (*cysE*) related to the feedback inhibition of cysteine synthesis was decreased in amended soils compared with that of acidic soils. Therefore, the change of relative abundance of functional genes in the nitrogen and sulfur cycle indicated that the shift of microbial community structure and function in HLB-infected citrus rhizoplane may have a forceful impact on soil microbial vitality and soil fertility reflected in the growth of the plant eventually.

The disease resistance and tolerance of plants are normally determined by heredity, but they could be modified by environmental factors, especially soil conditions and microorganisms (Trivedi et al., 2020; Thakur et al., 2021). Likewise, the native plant-associated microbial communities are synergistically affected by severe soil acidification and CLas infection in most citrus orchards in south China. This means that it is reasonable and feasible to bolster the stable and healthy plant-microbe associations to a certain extent by neutralizing the acidified soil around the roots of diseased citrus, thus promoting the root nutrient uptake and disease tolerance of citrus. Our research revealed that this practice can temporarily reduce the content of CLas pathogen in roots and boost diseased root vigor. Similar studies showed that applications of Ca, Mg, and B could alleviate the acidification of the soil, alter the phyllosphere and rhizosphere bacterial microbiome and reduce the HLB incidence in Gannan Navel Orange (Zhou et al., 2021). On the other hand, HLB-affected trees benefit from the additional application of essential nutrients in terms of growth and productivity; however, this effect is often inconclusive and unenduring (Morgan et al., 2016; da Silva et al., 2020; Shahzad et al., 2020; Atta et al., 2021). Thus, the CLas pathogen cannot be absolutely eliminated by improving the soil conditions, that is to say, the influence of HLB disease on citrus root-related microbial communities will not completely disappear.

The improvement of soil conditions seems to be beneficial to support the health of the whole citrus trees and might be able to slow HLB disease progression by influencing the resident microbiota, but it is unlikely that a single measure can prevent the occurrence and development of HLB disease. Therefore, many factors, such as disease severity, soil conditions, and community density of citrus psyllids, should be considered comprehensively when formulating control measures for HLB disease. It may be more effective to improve the effectiveness and sustainability of HLB disease management by integrating the approaches of acidic soil improvement, foliar and ground-applied essential nutrients, and inoculating beneficial plant microorganisms with insecticidal controls. On the horizon, there are prospects for utilizing pathogen-free citrus seedlings and breeding tolerant/resistant cultivars to combat HLB disease, because prevention is always a better choice than treatment afterward.

## Conclusion

The present study investigated the variations in soil properties and HLB-infected citrus root-associated microbial community structure and function in response to the lime application. The results indicated that liming is an effective managerial practice to mitigate soil acidity of citrus orchards in south China, which consequentially improved the vitality of citrus roots and reduced pathogen CLas relative concentration in roots. Additionally, this treatment strongly altered root endophytic microbial community diversity and structure, which is represented by the enrichment of beneficial microorganisms in roots; and it also recruited more functional genes that are involved in host-microbe interactions and nitrogen and sulfur metabolisms in the HLB-infected citrus rhizoplane, which are important in the microbiome-inhabiting plant root surfaces and the utilization of soil nutrients, respectively. These rhizoplane-enriched functional properties may subsequently benefit the plant's health and tolerance to HLB disease. However, this study concentrated only on the short-term responses of soil pH and plant-associated microbial communities after the application of soil amendments in acidic and HLB-infected soils. Whether liming has a persistent effect on soil pH and soil microbial communities or the likely duration of that effect would need further investigation. Overall, our study provides a novel insight for understanding the relationship between neutralization of acidified soil, citrus root-associated microbiota, and citrus tolerance to HLB.

## Data Availability Statement

The datasets presented in this study can be found in online repositories. The names of the repository/repositories and accession number(s) can be found in the article/Supplementary Material.

## Author Contributions

DQ and ShuaW conceived and designed the experiments. BL performed the experiment and data analysis. FF and ShutW gave their constructive criticism of an earlier version of the manuscript. YW and TH helped to revise and approved the final manuscript. All authors contributed to the article and approved the submitted version.

## Funding

This work was financially supported by grants from the China Agriculture Research System of MOF and MARA (CARS-27), the National Key R&D Program of China (2018YFD0201500), and the China Scholarship Council (CSC, 201903250116).

## Conflict of Interest

The authors declare that the research was conducted in the absence of any commercial or financial relationships that could be construed as a potential conflict of interest.

## Publisher's Note

All claims expressed in this article are solely those of the authors and do not necessarily represent those of their affiliated organizations, or those of the publisher, the editors and the reviewers. Any product that may be evaluated in this article, or claim that may be made by its manufacturer, is not guaranteed or endorsed by the publisher.
