# Supplementary material for: Root-Associated Microbiota Response to Ecological Factors: Role of Soil Acidity in Enhancing Citrus Tolerance to Huanglongbing
Source: Front Plant Sci. 2022 Jul 15;13:937414. doi: 10.3389/fpls.2022.937414 (PMC9335078; doi:10.3389/fpls.2022.937414)

Supplementary Material

# Supplementary Figures and Tables

## Supplementary Table

Table S1. Summary of the bacterial diversity and richness indices of endophyte in HLB-affected citrus root form acidified soils (SCK) and lime-amended soil (SLL and SHL).

| **Index** | **SCK** | **SLL** | **SHL** |
| --- | --- | --- | --- |
| **OTU numbers** | 339.67 ± 24.44a | 541.33 ± 32.44b | 515.0 ± 9.33b |
| **Chao1** | 490.77 ± 41.14a | 639.62 ± 44.27b | 641.68 ± 36.27b |
| **ACE** | 533.52 ± 87.13a | 652.65 ± 42.56b | 660.97 ± 34.80b |
| **Shannon** | 2.60 ± 0.10a | 4.32 ± 0.08b | 4.48 ± 0.14b |
| **Simpson** | 0.17 ± 0.01b | 0.03 ± 0.004a | 0.02 ± 0.002a |

Different letters in the column represent significant differences between treatments by the Tukey’s HSD test (p < 0.05).

## Supplementary Figures

**Figure S1.** Principal-coordinate analysis (PCoA) based on bray_curtis distances for the 16S rRNA amplicon of bacterial endophytes in HLB-infected roots collected form acidified (SCK) or amended soils (SLL and SHL).


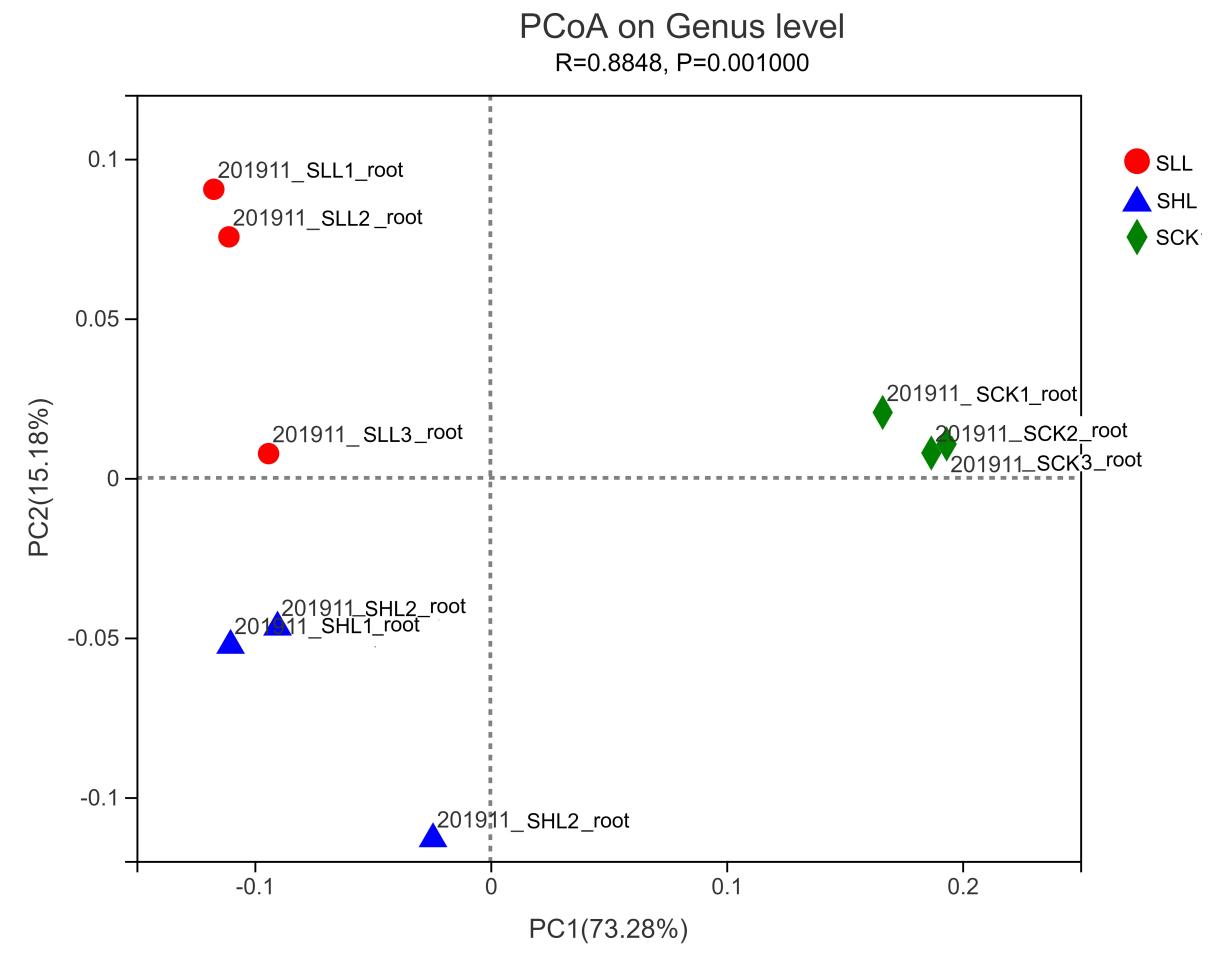


**Figure S2.** LEfSe analysis identifies different abundant bacterial taxa in HLB-infected root samples form SCK & SLL **(A)** and SCK & SHL **(B)** soils. The circles from inside to outside represent the taxonomic levels from phylum to genus. The small circle represents a taxon at that taxonomic level, and the diameter of the small circle represents the relative abundance. The colour of a taxon indicates that the relative abundance is significantly higher in the corresponding treatment (LDA > 4.0, P < 0.05), and the yellow colour indicates no significant difference. Only bacterial taxa with relative abundance > 0.1% are shown in the figure.


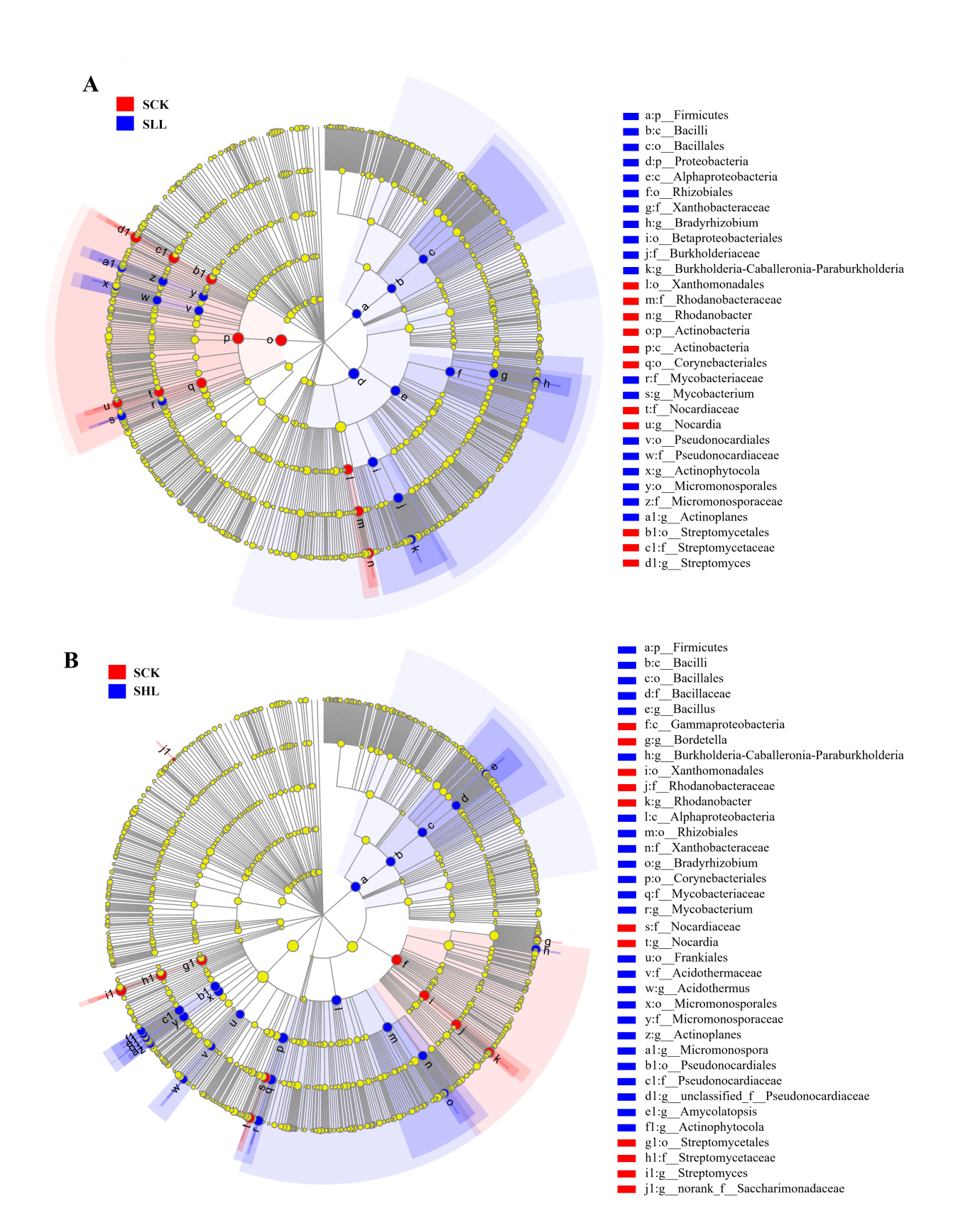


**Figure S3.** Changes of relative abundance of enzymes related to nitrogen metabolism **(A)** and sulfur metabolism **(B)** in HLB-infected citrus rhizoplane in different soils. SCK: soil without lime treatment; SLL: soil treated with low-level lime; and SHL: treated with high-level lime.


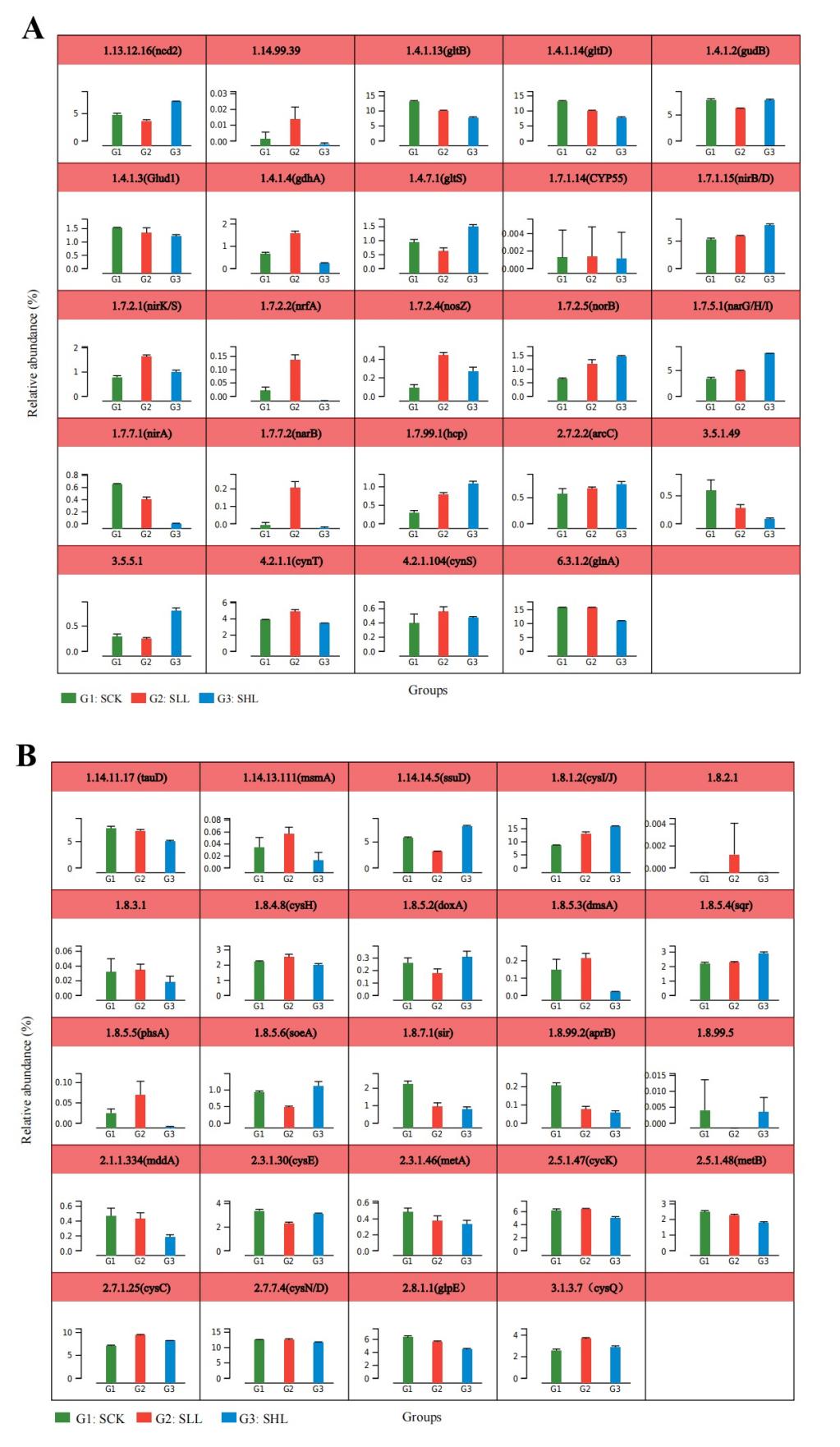

Supplement: Supplementary file 1 [file Data_Sheet_1.docx]
